# Management of Calcified Coronary Lesions—A Review of Plaque Modification Methods

**DOI:** 10.3390/jcm14238566

**Published:** 2025-12-03

**Authors:** Piotr Kałmucki, Paulina Skonieczna, Artur Baszko, Tomasz Siminiak

**Affiliations:** 1 Department of Cardiovascular Diseases Prevention, Poznan University of Medical Sciences, 61-701 Poznan, Poland; tsiminia@ump.edu.pl; 2 HCP Medical Centre, 61-485 Poznan, Poland; paulina.skonieczna@cmhcp.pl; 3 2nd Department of Cardiology, Poznan University of Medical Sciences, 61-485 Poznan, Poland; abaszko@ump.edu.pl

**Keywords:** percutaneous coronary intervention, coronary artery calcification, coronary artery atherectomy, intravascular imaging

## Abstract

Coronary artery disease remains the leading cause of cardiovascular morbidity and mortality worldwide, affecting millions of individuals each year. Coronary artery calcification is a common finding in patients with advanced atherosclerosis and represents an important determinant of procedural success during percutaneous coronary intervention. Severe calcifications are associated with increased procedural complexity and elevated complication rates due to challenging lesion preparation, suboptimal stent expansion, and less favorable long-term clinical results. This review summarizes the present understanding of vascular calcification mechanisms, discusses relevant diagnostic imaging modalities, and describes current plaque modification techniques used to optimize procedural outcomes. Methods such as rotational, orbital, and laser atherectomy, as well as specialized balloon technologies and intravascular lithotripsy, are discussed with regard to their mechanisms of action, clinical effectiveness, and safety profiles. Particular emphasis is placed on the integration of advanced imaging for precise lesion assessment, improved patient selection, and the use of combination strategies in complex cases. Finally, emerging technologies and future directions are highlighted, with the goal of enhancing procedural safety, device deliverability, and treatment outcomes in the evolving field of interventional cardiology.

## 1. Introduction

Cardiovascular diseases, including coronary artery disease (CAD), continue to represent the main cause of mortality worldwide, accounting for nearly one-third of all global fatalities [1]. The primary origin of coronary artery disease is atherosclerosis—a complex sequence of cellular mechanisms that drive plaque formation and gradual narrowing of the vascular lumen [2]. Coronary artery calcification represents an advanced manifestation of this process, reflecting both plaque burden and vascular degeneration, and acting as a robust predictor of adverse cardiovascular events [3]. Data from the large MESA cohort demonstrate that progression of coronary artery calcification is strongly associated with an increased risk of acute coronary syndromes and cardiac arrest [4]. At the cellular level, coronary artery calcification arises from a complex interaction among endothelial injury, lipid accumulation, and chronic inflammation. Activated macrophages and inflammatory cytokines such as IL-6, TNF-α, and IL-1β stimulate vascular smooth muscle cells to shift toward osteogenic differentiation through pathways involving RUNX2 and Wnt/β-catenin signaling. Simultaneously, various forms of programmed cell death, including apoptosis and autophagy, contribute to the release of matrix vesicles and dysmorphic calcium deposition within the arterial wall, ultimately promoting plaque mineralization and progression of atherosclerosis [5,6]. Intimal calcifications, most common in CAD, are considered to be particularly associated with age, diabetes mellitus, dyslipidemia, smoking, and systemic inflammation [3]. The assessment of plaque calcification severity severity may involve a non-invasive coronary artery calcification score (Agatston score) derived from computed tomography (CT), as well as fluoroscopic and intravascular imaging (IVI) performed during percutaneous coronary intervention (PCI). Precise quantification of coronary artery calcification prior to PCI enables tailored procedural planning, selection of the most suitable plaque-modification strategy, and optimization of clinical outcomes. IVI modalities such as intravascular ultrasound (IVUS) and optical coherence tomography (OCT) facilitate the assessment of calcium features and guide the choice of appropriate calcium debulking techniques [7]. Adequate lesion preparation reduces the risk of stent underexpansion, malapposition, and related complications such as thrombosis or target lesion failure, improving both procedural safety and long-term vessel patency [8,9,10].

## 2. Lesion Assessment

Coronary artery calcification (CAC) can serve as an initial diagnostic indicator of coronary atherosclerosis in patients with a low likelihood of obstructive coronary artery disease. According to the latest recommendations of the European Society of Cardiology, the absence of CAC carries a high negative predictive value, allowing clinicians to safely defer further investigation in patients with a calcium score of zero [11]. Non-invasive evaluation of the coronary artery calcium score (CACS) is commonly performed using ECG-gated cardiac computed tomography protocols without contrast enhancement, but it can also be achieved using a patient’s previous non-synchronized thoracic CT scans performed for non-cardiac indications; the results correlate reasonably well with those of dedicated cardiac CT imaging [12,13]. CAC quantification is based on calcium mass and volume or, more frequently, on the Agatston scoring system, which calculates the area of all pixels with a density above 130 Hounsfield units for each coronary artery and combines them into a cumulative index of the calcium burden. Calculation requires dedicated software but still depends on manual selection of coronary calcifications, a time-consuming process that necessitates operator training and experience [14]. Automated analysis using artificial intelligence and refined post-processing algorithms are expected to streamline this workflow. Subsequent contrast administration during ECG-gated cardiac CT angiography protocols enables further comprehensive evaluation of non-calcified plaques and luminal stenosis and is indicated in patients with a low or moderate probability of obstructive coronary artery disease [11]. However, a reliable CCTA assessment of luminal narrowing in severely calcified lesions can be challenging, especially in the presence of blooming artifacts, which distort the true lumen size and lead to an overestimation of the stenosis [15]. Functional lesion assessment with CT-derived fractional flow reserve (FFR-CT), which incorporates computational flow modeling, can improve diagnostic confidence. Although FFR-CT maintains excellent sensitivity in patients with a heavy coronary calcium burden, the considerable number of false-positive results still compromises the overall specificity in this group of patients [16]. Suboptimal image quality, patient-related motion, and variability in acquisition parameters may affect the diagnostic accuracy of CCTA and the reliability of FFR-CT calculations [15]. Modern technologies such as ultrahigh-resolution photon-counting CT, which transduces each detected photon into an electrical response and generates high-quality images, may increase the accuracy of both CAC scoring [17] and CCTA [18,19], but their poor availability significantly limits their use in everyday practice. Consequently, invasive coronary angiography combined with intravascular imaging and coronary physiologic assessment remains the gold-standard method for evaluation of the severity of artery stenosis in patients with calcified coronary lesions.

In fluoroscopy, calcified plaques appear as linear radiopaque or tramline-like densities that are clearly distinguishable even before contrast administration and without cardiac motion. When calcific deposits are observed bilaterally along the vessel wall and extend over a cumulative length of at least 15 mm, they indicate a high calcium burden [10]. This allows a rough classification of the severity of calcification (mild, moderate, or severe) [7,8]. Although highly specific, fluoroscopy lacks the sensitivity to precisely evaluate calcium depth, circumferential arc, and lesion length and therefore cannot effectively guide procedural decision making during PCI.

Accurate assessment of calcified coronary lesions requires the use of intravascular imaging. Intravascular ultrasound (IVUS) is a catheter-based modality that generates cross-sectional images of the vessel wall using ultrasound, allowing real-time evaluation of lumen, plaque, and calcium morphology. IVUS identifies calcifications as bright, hyperechoic regions with acoustic shadowing, enabling quick and appropriate measurement of calcific lesions: calcium arc, length, and location (superficial vs. deep). The extent of shadowing and the presence of reverberation artifacts on IVUS images make it possible to assess the estimated calcium thickness (reverberations suggest <0.5 mm, while complete shadowing suggests >1 mm calcium thickness). Moreover, IVUS provides an intraprocedural evaluation of the results of percutaneous coronary angioplasty, allowing confirmation of the proper modification of the plaque and correct stent apposition. The main limitations of IVUS are the relatively large catheter (approximately 3.2 F), which may not be able to cross highly calcified, narrow lesions, and poor spatial resolution, typically 100–150 μm for classical probes [20]. Recently emerged high-definition intravascular ultrasound (HD-IVUS) probes (OptiCross, Boston Scientific, Marlborough, MA, USA and TrueVision, Insight Lifetech, Shenzhen, Guangdong, China) offer a higher image resolution that enables a more detailed evaluation of atherosclerotic plaques. However, due to the ultrasound-based nature of this technology, the acoustic shadow behind calcium deposits still partially limits the complete evaluation of calcified lesions [21].

Optical coherence tomography (OCT) is an intracoronary imaging technique that uses near-infrared light to produce high-resolution (10–15 μm) cross-sectional images of the vessel microstructure. In OCT, calcium is identifiable as a heterogeneous low-signal region with well-defined margins, minimal backscatter, deep light penetration, and no associated shadowing. OCT excels in delineating superficial calcium, measuring its thickness, arc, and longitudinal extent [21]. Simple OCT scoring systems such as the “rule of 5” (which considers an arc >50% of vessel circumference, thickness >0.5 mm, and length >5 mm [22]) and, more recently, the “rule of 3” (which stands for 360° arc, thickness >0.3 mm, and length >3 mm) both correlate strongly with the likelihood of stent underexpansion [23]. Along with assessing plaque morphology, OCT enables real-time visualization of periprocedural plaque modification—such as fracture lines or tissue disruption—and allows post-PCI confirmation of optimal stent deployment (e.g., minimum stent area, stent apposition, and no edge dissections). Based on these features, Shlofmitz et al. developed the MLD MAX algorithm to streamline the workflow during OCT-guided PCI [24]. The properties of IVUS and OCT are compared in Table 1 below.

The initial stage of coronary artery calcification involves microscopic deposits, typically observed in areas of intimal thickening, with dimensions ranging between 0.5 and 15 μm. As calcification progresses, plaques often develop extensive calcium layers spanning more than one quadrant of the vessel wall. Mechanical disruption of these calcified plates may result in the emergence of very difficult-to-treat calcified nodules [26]. Certain morphological features of calcified atherosclerotic plaques have been shown to significantly increase the risk of stent underexpansion and future in-stent restenosis. Consequently, dedicated scoring systems have been developed to evaluate such lesions and guide the need for plaque modification prior to stent implantation. Fujino et al. [27] proposed an OCT-based rating system, recently revised by Sato et al. [23], and similarly, Zhang et al. [28] established IVUS-derived criteria. Both scoring systems have been incorporated into the Expert Consensus Statement on the Management of Calcified Coronary Lesions [10] and are summarized in Table 2.

A final score of ≥2 points on the OCT scale [23] or ≥2 points on the IVUS scale indicates the need for appropriate plaque preparation with advanced calcium modification techniques to enable optimal stent deployment [28].

**Figure 1:**
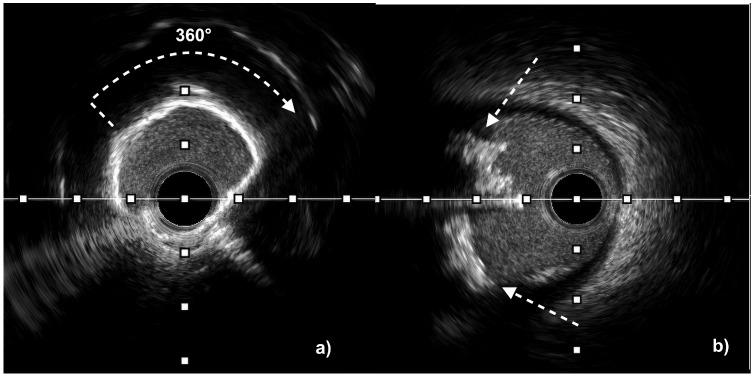
High-definition intravascular ultrasound (HD-IVUS) images illustrating severe coronary calcifications: (**a**) a circumferential (360°) calcified lesion appearing as a bright ring with an acoustic shadow behind it (white arrow); and (**b**) an eruptive calcified nodule appearing as an irregular, protruding calcified mass extending into the vessel lumen with an acoustic shadow (white arrows indicate the proximal and distal margins of the calcified nodule).

**Figure 2:**
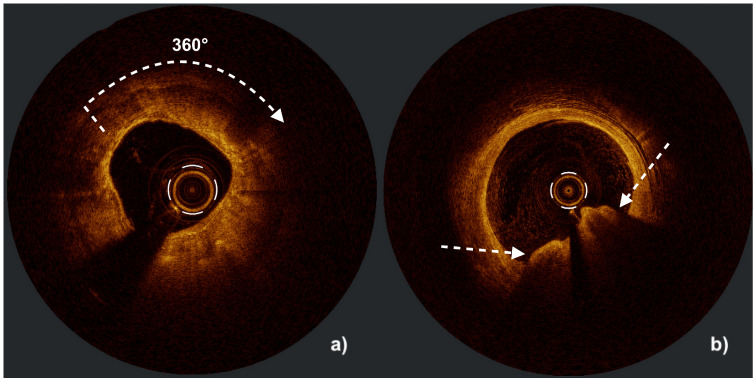
Optical coherence tomography (OCT) images illustrating severe coronary calcifications: (**a**) a circumferential (360°) calcified lesion appearing as a heterogeneous, low-signal region with well-defined borders, minimal backscatter, deep light penetration, and no shadowing (white arrow); (**b**) an eruptive calcified nodule visualized as a heterogeneous, low-signal mass with similar optical characteristics, protruding into the vessel lumen (white arrows indicate the margins of the calcified nodule). Figure courtesy of Professor Maciej Lesiak and Sylwia Iwańczyk, Ph.D.

A calcified nodule (CN) is a protruding mass of calcium within the coronary lumen, typically overlying a heavily calcified plaque; such nodules are often present in the proximal and medial segments of the right coronary artery, which are exposed to high flexural stress, or in lipid-rich and necrotic-core-dominant areas, including the left main bifurcation and proximal parts of the epicardial arteries [29]. CNs tend to occur more frequently in older individuals, women, patients with comorbidities such as diabetes mellitus or history of previous coronary artery bypass grafting (CABG), and also in those receiving chronic hemodialysis [30]. CNs are histologically categorized as eruptive (with fibrous cap disruption, an irregular surface, and thrombus formation) or non-eruptive (with a smooth surface and an intact cap) [31]. CNs are associated with an elevated risk of long-term cardiovascular events and poor invasive treatment results due to suboptimal stent expansion and a high percentage of in-stent restenosis. Non-eruptive calcium nodules are less susceptible to balloon deformation and require accurate, often multi-stage lesion modification to avoid stent underexpansion. On the other hand, eruptive CNs may allow more symmetrical acute stent deployment, but they are linked to worse long-term outcomes due to early in-stent restenosis caused by the reprotrusion of nodular debris into the vessel lumen [10]. Intraprocedural differentiation of CN subtypes is achievable using IVI—particularly with HD-IVUS or more precisely with OCT because of its superior resolution. Examples of concentric calcifications and eruptive calcified nodules obtained in HD-IVUS and OCT imaging are presented below in Figure 1 and Figure 2.

## 3. Modification Methods for Calcified Plaques

Percutaneous treatment of heavily calcified coronary lesions remains challenging, with increased periprocedural risk, elevated incidence of early complications, and suboptimal success rates. The selection of appropriate calcium modification techniques seems to be crucial for effective lesion preparation, optimal stent deployment, and avoidance of adverse events.

### 3.1. SC and NC Balloons

Semi-compliant balloons are thin-wall angioplasty devices constructed from soft, flexible polymers. They are commonly used for lesion predilatation in non-calcified coronary stenoses; for plain old balloon angioplasty (POBA); or as carriers for antiproliferative agents in drug-coated balloons. Due to their trackability and flexibility, they are advantageous for crossing tight or tortuous lesions. However, when deployed against heavily calcified plaques under high inflation pressures, SC balloons may expand non-uniformly, widening at the proximal and distal edge while remaining constricted at the lesion site—often referred to as a “dog-bone” or “dumbbell” effect. This phenomenon not only indicates insufficient plaque modification but also has the potential to cause serious complications such as balloon rupture, vessel perforation, or dissection, especially at the edges of the balloon [32].

Non-compliant balloons are composed of thick, rigid walls of durable polymers. These balloons exhibit limited expansion in response to increasing inflation pressure, resulting in a more controlled and predictable diameter. This feature allows the application of high radial force against the lesion with minimal risk of overexpansion, making these balloons a first-line tool for predilatation of calcified lesions, especially when intravascular imaging or advanced plaque modification devices are not insertable through a tight coronary stenosis. However, in the presence of asymmetric, sharp, or deep calcifications, even NC balloons may display incomplete expansion, a “dog-bone” effect during inflation, or even rupture at very high pressures [32].

Clinical data suggest that even with relatively simple lesions, the use of non-compliant balloons for lesion preparation results in more uniform expansion and better procedural outcomes than the use of semi-compliant alternatives. These findings have contributed to a paradigm shift in predilatation strategies, favoring high-pressure, non-compliant balloon inflation for optimal stent bed preparation, regardless of the lesion complexity [33].

### 3.2. Super-High-Pressure Non-Compliant Balloons

Super-high-pressure non-compliant balloons, such as the OPN (SIS Medical, Frauenfeld, Switzerland), are designed with a dual-layer architecture to withstand inflation pressures up to 35 atmospheres [34] and apply significant forces to modify severely resistant lesions, including undilatable de novo calcifications and in-stent restenoses. Their reinforced structure distributes pressure between the inner and outer layers, reducing the risk of vessel perforation in the event of balloon rupture. Super-high-pressure NC balloons are relatively easy to use and do not require additional operator training. However, due to their bulky design and relatively large crossing profile, adjunctive methods such as a “buddy wire” technique or guide extension catheter may be necessary to advance the device to the target site in case of a narrow, complex lesions [32]. Compared with the use of standard non-compliant balloons, the use of OPN devices for complex, non-dilatable lesions was associated with a significantly reduced residual diameter stenosis [35].

### 3.3. Scoring and Cutting Balloons

Scoring balloons are modified balloon devices equipped with an external nitinol net designed to focus contact force without provoking vessel wall pressure injury. Force transmission through nitinol struts generates controlled microfractures within the plaque and results in efficient disruption of calcium at lower inflation pressures. The structural design also provides anchoring during inflation, which helps prevent slippage and ensure stable balloon expansion. Because most scoring balloons (AngioSculpt EVO, Philips, Amsterdam, The Netherlands or NSE Alpha, B. Braun, Melsungen, Germany or Naviscore, iVascular, Barcelona, Spain) are based on semi-compliant platforms, they maintain favorable crossability and trackability, offering advantages in anatomies where bulky devices are difficult to deliver [36]. Different technology is applied in the Scoreflex (OrbusNeich, Hong Kong, China)—a NC balloon family, where a single nitinol wire and a segment of standard workhorse guidewire are used as scoring components [32]. A combination of hydrophilic and hydrophobic coatings reduces friction to improve the deliverability of the NC-based device to the lesion site [22].

Cutting balloons are specialized non-compliant angioplasty devices equipped with three or four longitudinal microsurgical blades. Cutting edges focus contact force to create controlled shallow incisions in fibrotic or calcified plaques at relatively low pressures, improving balloon expansion and facilitating optimal stent delivery [37]. Reduction of applied pressures limits the risk of uncontrolled dissection or vessel injury. The main factor preventing the widespread application of cutting balloons is their bulky profile, which gives them a poor ability to cross narrow lesions. Newer designs—such as the Wolverine balloon (Boston Scientific, Marlborough, MA, USA) —have improved deliverability through lower-profile shafts and more flexible distal tips [38]. In the COPS trial, patients with calcified plaques randomized to cutting balloon predilatation achieved a greater final minimum stent area than those treated with standard NC balloons [39]. Other studies comparing scoring and cutting balloons suggest better stent deliverability after cutting balloon predilatation, even in challenging anatomies [40]. The features of balloon-based devices are summarized in Table 3 presented below.

### 3.4. Coronary Atherectomy

Atherectomy is a percutaneous intervention designed to partially remove atherosclerotic plaque responsible for vascular obstruction. In contrast to angioplasty, which compresses the plaque into the arterial wall, this approach physically eliminates part of the lesion. Coronary atherectomy is an important modality for the modification of calcified lesions, especially in tight, balloon-uncrossable stenoses. Available atherectomy methods include rotational, orbital, and laser atherectomy.

#### 3.4.1. Rotational Atherectomy

Rotational atherectomy (RA), also known as rotablation, is one of the oldest and most established techniques to modify calcified coronary plaques. Despite its long history, it remains underused due to its procedural complexity and the relatively time-consuming operator training required.

The most popular ROTAPRO™ system (Boston Scientific, Marlborough, MA, USA) requires specialized 0.009″ guidewires, such as the ROTAWIRE™ or ROTAWIRE™ Drive, from a new family of guidewires with improved trackability. After the Rotawire is positioned distal to the lesion, the diamond-coated elliptical burr begins to spin over the wire at 140,000 to 180,000 rpm and, through a controlled “pecking motion”, progressively ablates the calcific plaque. A burr-to-artery ratio of approximately 0.6 is recommended [41,42], with smaller burrs (1.25–1.5 mm) favored for long or tortuous segments and larger ones reserved for proximal or aorto-ostial disease. Most procedures can be performed with burr sizes up to 1.75 mm via radial approach with a 6 Fr guiding catheter. Burrs of 2.0 mm diameter require a 7 Fr system or a sheathless radial approach, while the largest devices may need an 8 Fr guiding catheter and a subsequent femoral approach [43]. A continuous flush solution, typically composed of heparinized saline with vasodilators such as verapamil or nitrates, must be administered through the side port of the device to cool the turbine, prevent vasospasm, and reduce debris accumulation during the procedure [42].

Unlike balloon techniques, RA removes plaque by mechanical abrasion, reducing vessel wall barotrauma and the risk of deep dissections. The most frequent complication of RA (5–20%) is the coronary slow-flow phenomenon (CSFP), usually resulting from the accumulation of atherosclerotic debris in the distal coronary circulation. The incidence of this complication is related not only to anatomical features such as small vessel diameter, angulation, and atherosclerotic lesion length but also to procedural factors such as high burr to vessel ratio, long ablation runs [44], and excessive drops in rotational speed. Although current, less aggressive burr-to-vessel ratio recommendations have reduced the incidence of CSFP, operators should remain cautious about the possibility of this complication. The moment a flow drop is noticed, rotational atherectomy should be suspended until normal TIMI-3 flow is re-established [43]. ST-segment elevation on ECG monitoring often precedes the onset of CSFP and can be valuable for intraprocedural surveillance during rotational atherectomy. Ensuring pre-procedural hydration and maintaining systolic blood pressure above 100 mmHg are important maneuvers in the prevention of CSFP [45]. Although rare, coronary artery perforations (1–2% of cases) [46,47] remain a potentially fatal adverse event of RA. Increased vigilance for perforation is required, especially when procedures are performed on eccentric calcifications—such as calcified nodules—or within narrow, tortuous coronary vessels [45]. Another major complication is burr entrapment, reported in 0.4–0.8% of cases. It can arise from anatomical angulation or the so-called “Kokesi phenomenon”, where the burr slips behind the lesion and becomes stuck due to its fusiform silhouette [48]. In some cases, this complication requires surgical intervention and coronary artery bypass grafting. Experienced operators emphasize the importance of monitoring early signs of rising resistance, such as alterations in ablation sound pitch and fluctuations in rotational speed, as prompt recognition and response can prevent device lodging, vessel injury, or other serious complications [42].

The ROTAPRO™ rotablation system (Boston Scientific, Marlborough, MA, USA), being equipped with a diamond coating only on the front half of the spinning burr, is a typical “front cutting device”. Due to the mechanism of action, it is especially effective in treating very tight or even balloon-uncrossable calcified lesions. In moderate stenoses, eccentric lesions, and calcified nodules, the use of RA, although possible, is less effective. A spinning burr can “shave” the calcium surface and even cause cracking in calcific nodules, but techniques such as OA or IVL tend to be more effective in such cases [49].

A recent advancement in RA technology, the FireRaptor System (MicroPort Rota Pace, Shanghai, China), features an eccentric and fully diamond-coated burr. This property allows the FireRaptor System, unlike the classic rotablation systems, to perform bidirectional plaque modification. Forward and reverse device movement not only decreases the risk of burr entrapment but also enhances cutting efficiency. In addition, adjustable rotational speed allows operators to tailor the ablation diameter to the size of the vessel and the characteristics of the lesion, reducing the need for multiple device exchanges [50].

#### 3.4.2. Orbital Atherectomy

Orbital atherectomy (OA) is another technique within the atherectomy family. The Diamondback 360^®^ Coronary Orbital Atherectomy System (Abbott Cardiovascular, Chicago, IL, USA) features a diamond-coated, eccentrically mounted 1.25 mm crown that rotates over a specialized 0.012″ ViperWire with a 0.014″ tip. The mechanism of OA relies on centrifugal force generated by the spinning crown, which orbits as it advances through a coronary artery and preferentially ablates calcified tissue while displacing softer structures. This unique mechanism of action allows continuous blood flow during atheroablation and generates very small, submicron debris (~2 μm), which passes safely through the microcirculation, resulting in a low risk of distal embolization and the slow-flow phenomenon [51]. The system incorporates single “one-size-fits-all” 1.25-mm crown, that can be delivered through a 6 Fr guide catheter. Changes in rotational speed (from 80,000 rpm to 120,000 rpm) translate into an increased sanding radius, thereby enabling treatment of different-sized vessels from 2.5 to 4 mm in diameter [52]. The procedure is aided by the infusion of the specific ViperSlide lubricant to reduce friction between the crown and vessel walls. Lesion preparation typically begins at the lower rotational speed, with careful advancement at 1 mm/s and no forward force applied. The operator is encouraged to monitor the acoustic feedback of the spinning crown to evaluate engagement with the lesion. Each run should be slow and limited to a maximum time of 30 s, while the total duration of atherectomy should not exceed 5 min due to expected device wear, as specified by the manufacturer [53]. The incidence of complications with orbital atherectomy is comparable to that observed with rotational atherectomy [54].

#### 3.4.3. Laser Atherectomy

Excimer laser coronary atherectomy (ELCA) is an atherectomy modality that uses high-energy ultraviolet light to ablate atherosclerotic tissue. The most popular system, the CVX-300 Laser Excimer System (Philips, Amsterdam, The Netherlands), generates short pulses at a wavelength of 308 nm by passing an electrical discharge through a rare gas–halogen mixture, producing excimer molecules that emit photons. Lesion modification occurs through combined photochemical bond disruption, photomechanical shockwave generation, and localized photothermal effect. This triple-modality action enables precise plaque ablation with minimal thermal injury to surrounding tissue and decreased formation of particulate debris (predominantly microfragments < 10 μm), thereby reducing the risk of distal embolization and no-reflow phenomenon [55].

A key advantage of ELCA over rotational or orbital atherectomy is its compatibility with any standard 0.014″ coronary guidewire, which is typically easier to advance across complex lesions than dedicated systems such as Rotawire or ViperWire. Catheters are available in various sizes ranging from 0.9 to 2.0 mm and should not exceed two-thirds of the vessel’s reference diameter. The most common 0.9 mm device is deliverable through a 6 Fr guide catheter, facilitating the ELCA procedure from radial access [56]. Device operation requires heparinized saline or contrast flushing to prevent bubble formation. Each 10-s activation is followed by a 5-s pause, repeated until the lesion is crossed or adequately modified for balloon passage or expansion [57]. For safety, the patient and all personnel must wear protective tinted eyewear to prevent retinal injury from exposure to UV light.

ELCA can be applied to native calcified lesions; balloon-uncrossable stenoses; chronic total occlusions; and in-stent restenoses (ISRs), especially with significant peri-stent calcifications [58]. Clinical outcome data from ELCA studies regarding ISR lesions showed no significant effect on stent structural integrity or polymer release [59]. Another advantage of ELCA is the ability to use side branch wire protection during treatment of resistant coronary bifurcation lesions [10]. In certain heavily calcified lesions, when ROTAWIRE™ advancement is not possible, ELCA may be applied first to create a channel that facilitates wire passage, allowing RA to be performed and the procedure to be completed successfully in what is known as the “RASER” technique [60]. This complex intervention offers a valuable bailout option for patients with lesions resistant to conventional PCI. However, due to the higher baseline and periprocedural risk, its introduction should be guided by multi-disciplinary Heart Team consultation and patient input [61].

### 3.5. Intravascular Lithotripsy

Intravascular lithotripsy (IVL) is one of the newer calcium modification techniques, which delivers pulsatile acoustic pressure waves from an external power source via spherical emitters integrated into a semi-compliant balloon. The monorail device is advanced to the lesion site over the standard coronary 0.014″ guidewire. The system applies low-pressure balloon inflation—typically around 4 atmospheres—ensuring vessel wall contact while minimizing barotrauma. Once the balloon is apposed to the vessel walls, a controlled electrical discharge within the fluid-filled balloon generates rapidly expanding vapor bubbles. Their collapse emits circumferential shockwaves that traverse soft tissue without damage but produce focal mechanical stress when encountering calcific deposits. The peak wave pressure is equivalent to approximately 50 atmospheres [62]. The targeted stress leads to multiplane microfractures in both the superficial and deep layers of calcium, improving vessel compliance. The shockwaves are generated at a consistent frequency of one pulse per second and delivered in sequences of ten, with a maximum of 120 pulses available in the new C2+ Generation Shockwave System (Shockwave Medical, Santa Clara, CA, USA). The integrated balloon serves multiple purposes: it stabilizes the system during energy delivery, facilitates coupling of acoustic energy through fluid–tissue impedance matching, and provides thermal protection to vascular structures and device components by dispersing generated heat [62]. Given the IVL balloon’s limited length of 12 mm and the minimum diameter of 2.5 mm, small vessels cannot be treated with this device, whereas long, calcified segments require sequential inflations. Another limitation of IVL is its relatively bulky profile, which can hinder delivery in tight or tortuous lesions. In such cases, auxiliary techniques or devices—such as guide extensions or prior atherectomy—may be required [63].

**Figure 3:**
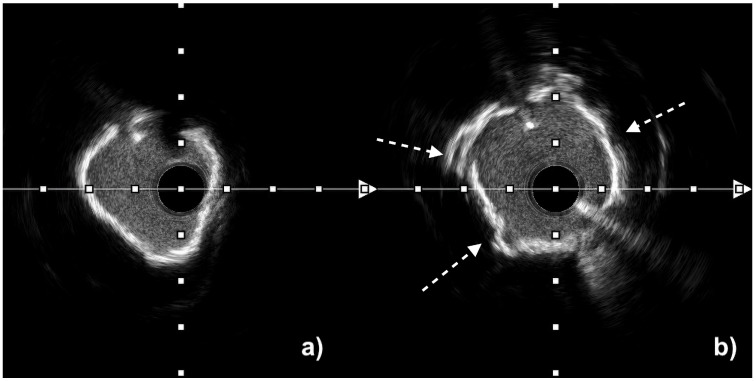
Intravascular ultrasound (IVUS) images of a severely calcified coronary lesion with circumferential (360°) calcification before (**a**) and after (**b**) intravascular lithotripsy (IVL) using the Shockwave C2+ catheter (Shockwave Medical, USA), demonstrating an increase in minimal lumen area and multiple calcium fractures (indicated by white arrows).

**Figure 4:**
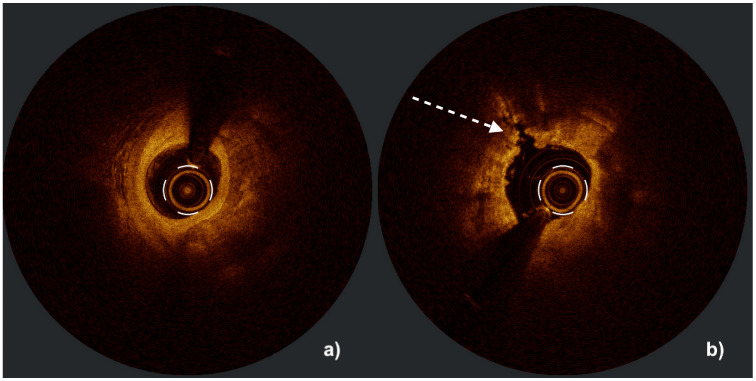
Optical coherence tomography (OCT) images of a severely calcified coronary lesion with circumferential (360°) calcification before (**a**) and after (**b**) plaque modification, demonstrating an increase in minimal lumen area and a calcium fracture (indicated by a white arrow). Figure courtesy of Professor Maciej Lesiak and Sylwia Iwańczyk, Ph.D.

IVL is highly effective in circumferential calcium lesions, where acoustic pressure waves can disrupt concentric calcified rings and restore vessel compliance. Clinically, its key advantage is also observed in the treatment of eccentric calcifications and calcified nodules, where conventional techniques—such as high-pressure non-compliant, scoring, or cutting balloons—carry an elevated risk of arterial dissection. In these challenging morphologies, rotational atherectomy is further limited by wire bias, often resulting in superficial “shaving” or lesion guttering rather than sufficient calcium modification. In contrast, IVL catheters selectively deliver acoustic energy toward dense calcific deposits while sparing healthy soft vessel wall regions, enabling safer and more uniform lesion preparation [64]. Figure 3 and Figure 4 presented below illustrate the effect of IVL in circumferential calcium lesions visualized with IVUS and OCT. IVL has also been successfully used for managing underexpanded stents, with evidence from registries showing significant increases in stent area and luminal gain [65]. Unlike RA or OA, the IVL catheter allows the placement of a side-branch protection wire during pulse delivery, making it particularly suitable for the treatment of coronary bifurcation lesions [66]. Due to its safety profile and lower risk of complications, IVL is also preferred over atherectomy in balloon-crossable calcified aorto-ostial lesions [10].

DISRUPT CAD III, a large prospective study involving 384 patients with severe coronary calcification, assessed the procedural success of IVL at over 92%, with perforation and slow-flow rates of 0.3% and 0.5% respectively [67]. IVL’s safety profile, with a minimal incidence of adverse events, was later confirmed in other studies, also including patients with acute coronary syndromes [68,69].

Driven by increasing clinical demand, next-generation devices based on established mechanisms of action are being developed and introduced worldwide. Several new IVL platforms are currently undergoing clinical evaluation (Abbott Cardiovascular, Chicago, IL, USA; Lepumedical, Beijing, China; Shunmei, Shenzhen, China), including the Sola system (FastWave Medical, Minneapolis, MN, USA; IDE trial starts in mid-2026), the first laser-based IVL platform with a single movable optical transmitter, and the Javelin Coronary IVL system (Shockwave Medical, Santa Clara, CA, USA), a single distal emitter device that enables forward calcium modification beyond the catheter tip and facilitates treatment of tight, non-crossable lesions [70]. The features of the described methods for modification of calcified lesions are summarized in Table 4 below.

The latest approach is a slightly different procedure called Hertz contact intravascular lithotripsy (HC-IVL). The technique is based on the Hertzian contact stress principle, where the force concentrated at small contact points generates sufficient stress to fracture rigid structures while preserving the surrounding soft tissues. The system consists of a semi-compliant balloon catheter with multiple rows of metallic hemispheres. When the balloon is inflated, these hemispheres apply discrete, high-intensity contact forces to the calcified plaque, producing controlled fractures without the need for any external energy source, which significantly reduces procedural complexity. The semi-compliant platform of HC-IVL provides improved deliverability, especially compared to standard IVL devices. Moreover, this design enables treatment of various lesion morphologies, adjusting to the size of the vessel during inflation. Repeated HC-IVL inflations allow effective management of long segments, including eccentric and concentric calcification, while maintaining a favorable safety profile [74].

LithiX™ HC-IVL (Elixir Medical Corporation, Milpitas, CA, USA) was assessed in the PINNACLE I trial, a prospective, multicenter single-arm study that included 60 patients with moderately to severely calcified coronary lesions. A very high clinical success rate was observed for the combined primary safety and efficacy endpoint (98.3%), along with 100% angiographic success and residual diameter stenosis <30% in all lesions. The target lesion failure rate was 1.7%, with a single perioperative non-Q-wave myocardial infarction. No cases of severe dissection, perforation, or abrupt closure of the vessel were observed [75,76].

## 4. Conclusions

Coronary artery calcification remains a major determinant of procedural success and long-term clinical outcomes in percutaneous coronary intervention. The choice of a calcium modification technique should be guided by intravascular imaging findings, lesion morphology, and operator expertise and experience. Despite significant progress, evidence from randomized trials comparing different calcium modification strategies is still limited. Future studies are required to establish standardized algorithms that integrate multimodal imaging and combination therapies to optimize stent expansion and improve patient prognosis in complex coronary disease with advanced atherosclerosis.

## Figures and Tables

**Table 1:** Comparison between IVUS and OCT, adapted from Koganti et al. [25].

Aspect	IVUS	OCT
Imaging modality	Ultrasound	Near-infrared light
Axial resolution	100–150 μm (20–40 MHz IVUS probes) 20–60 μm (60 MHz HD-IVUS probes)	10–20 μm
Lateral resolution	200 μm	20 μm
Tissue penetration depth	>5 mm	1–2 mm
Need for blood clearance	No	Yes
Need for contrast injection	No	Yes (may be replaced by dextran)
Catheter size	Up to 3.2 Fr	2.7 Fr
Assessment of plaque morphology	Moderate resolution for plaque assessment	Superior fine detail (fibrous cap, lipid core, thrombus)
Calcium assessment	Semi-quantitative (measures calcium length and arc but only estimates thickness) Microcalcifications not assessable Difficulty in differentiating between eruptive and non-eruptive CNs	Quantitative (measures calcium length, arc, and thickness) Microcalcifications assessable Accurate differentiation between eruptive and non-eruptive CNs Medial calcifications difficult to assess
Evaluation of vessel remodeling	Yes	Limited
Utility in aorto-ostial lesions	Preferred (guideline-recommended)	Difficult, less preferred
Stent expansion assessment	Reliable, deeper wall visualization	Highly precise but limited by EEM ^1^ visibility
Detection of malapposition/dissection	Possible, less sensitive	Superior due to resolution

^1^ EEM—external elastic membrane.

**Table 2:** Calcified coronary lesion scoring systems for OCT and IVUS, reproduced from Sato et al. [23] and Jurado-Román et al. [22].

OCT			IVUS		
Calcium arc	<360°	0	Calcium arc	≤270°	0
	360°	1		>270° and >5 mm length	1
Calcium thickness	≤0.3 mm	0	Calcified nodule	No	0
	>0.3 mm	1		Yes	1
Length of calcium > 270°	≤3 mm	0	Coronary artery diameter	≥3.5 mm	0
	>3 mm	1		<3.5 mm	1

**Table 3:** Comparison of balloon-based devices used in Percutaneous Coronary Interventions (PCI).

Aspect	Semi-Compliant Balloon	Non-Compliant Balloon	Super-High-Pressure NC Balloon	Scoring Balloon	Cutting Balloon
Device design	Thin, extensible single-layer balloon	Thick, non-extensible single-layer balloon	Double-layer, reinforced non-compliant balloon	SC or NC balloon with nitinol scoring elements	NC balloon with longitudinal microblades
Primary mechanism of action	Uniform plaque compression and vessel stretching at low pressures	Focal plaque compression, predictable expansion	Modification of fibro-calcific lesions with barostatic force	Controlled scores created by nitinol elements	Tissue microincisions from microblades
Typical pressure range (NP-RBP)	∼6–14 atm	∼12–22 atm	∼10–35 atm	∼8–20 atm	∼6–12 atm
Main indications	Narrow lesions, predilatation in mild disease	Predilatation, postdilatation, moderately calcified lesions	Heavily calcified lesions, resistant underexpanded stents	Fibrotic/moderately calcified lesions, ISR	Focal fibro-calcific lesions, ISR
Advantages	Flexibility, deliverability, low crossing profile	Predictable diameter; good stent expansion	Effective for resistant lesions; strong radial force	Reduced slippage, controlled plaque modification	Precise plaque incision; low risk of arterial wall barotrauma
Limitations	Ineffective in severe calcification, risk of over-dilatation	Low efficiency in severe calcifications, risk of dissections	Bulky, higher perforation risk if oversized	Bulky, difficult to deliver, costly	Bulky, difficult to deliver, costly
Deliverability	+	+	- -	-	- -
Calcified lesions	-	+	++	++	++
Calcific nodules	-	-	- -	+	+
Fibrotic lesions	-	+/-	-	+	++
Stent underexpansion	-	+	++	+	+
ISR	-	+	+	++	++

Symbol definitions: ++ excellent/very good performance; + good performance; +/- moderate performance; - poor performance; - - very poor performance. Abbreviations: SC—semi-compliant; NC—non-compliant, NP—nominal pressure; RBP—rated burst pressure; ISR—in-stent restenosis.

**Table 4:** Comparison of calcified lesion modification methods, adapted from Russo et al. [71], Sasi et al. [72], and Yeung et al. [73].

Aspect	IVL	RA	OA	ELCA
Mechanism of action	Lithotripsy via acoustic pressure waves	Atheroablation via front abrasion	Atheroablation via sanding	Photoablation (light, acoustic pressure waves, cavitation microbubbles)
Guidewire	Elective 0.014″ wire	Dedicated 0.009″/0.014″ tip wire	Dedicated 0.012″/0.014″ tip wire	Elective 0.014″ wire
Device size	2.5–4.0 mm × 12 mm	1.25–2.5 mm (5–8 Fr)	One crown size 1.25 mm (6 Fr)	0.9–2.0 mm with concentric and eccentric tip designs
Course of action	Forward and backward On the balloon’s adhesion surface	Forward only Outside curve only	Forward and backward Outside and inside curve	Forward only
Effect of wire bias	Independent	Dependent	Less dependent	Limited by vessel curvature (UV light does not deflect)
Side branch protection	Yes	No	No	Yes
Distal embolization	No or very low risk of no/slow reflow	Higher risk of no/slow reflow	Medium risk of no/slow reflow	Very low risk of no/slow reflow
Perforation	Low <1%	Up to 1.5%	Up to 1.8%	1.5–2%
Effect on calcium	Affects superficial and deep calcium	Affects only superficial calcium	Affects only superficial calcium	Different effects on superficial and deep calcium

## Data Availability

Not applicable.
